# Surface charge of Merkel cell polyomavirus small T antigen determines cell transformation through allosteric FBW7 WD40 domain targeting

**DOI:** 10.1038/s41389-020-0235-y

**Published:** 2020-05-19

**Authors:** Nnenna Nwogu, Luz E. Ortiz, Hyun Jin Kwun

**Affiliations:** Department of Microbiology and Immunology, Pennsylvania State University College of Medicine, Hershey, PA USA

**Keywords:** Virology, Skin cancer

## Abstract

Merkel cell polyomavirus (MCV) small T (sT) is the main oncoprotein in Merkel cell carcinoma (MCC) development. A unique domain of sT, LT stabilization domain (LSD), has been reported to bind and inactivate multiple SCF (Skp1-Cullin-F-box) E3 ligases. These interactions impede the turnover of MCV large T (LT) antigen and cellular oncoproteins such as c-Myc and cyclin E, thereby promoting viral replication and cell transformation. However, it is currently unclear how this LSD region contributes to multiple transforming activities of sT. Structural docking simulation of sT and F-box and WD repeat domain-containing 7 (FBW7) revealed a novel allosteric interaction between sT and FBW7 WD40 domain. This model is supported by experimental evidence confirming that charge engineering in the LSD alters sT-WD40 binding. Specifically, loss of net positive charge in the LSD prevents sT-FBW7 binding by abrogating the electrostatic interaction, thus impeding inhibition of FBW7 by sT. Furthermore, positively charged mutations in the LSD significantly restored the sT function and its ability to transform rodent fibroblast cells. We infer that the surface charge of sT is a major determinant for targeting E3 ligases, which leads to sT-induced cell transformation, an observation that could be used to develop targeted therapeutics for MCC.

## Introduction

E3 ubiquitin ligases are a large family of proteins engaged in the proteolytic degradation of many target proteins, regulating their turnover and activity. The Skp1, Cullin, F-box containing (SCF) complex is a key player in cell cycle regulation, and has been shown to be a major moderator of essential proteins involved in cell cycle progression^1^. E3 ligase substrate recognition and binding are mediated by F-box proteins, which function as a scaffold for protein-protein interaction and link substrates to the SCF complex. The F-box protein family is classified based on the specific substrate recognition domains identified within the F-box motif. These classes include: FBWX, which contains tryptophan (W)-aspartic acid (D) dipeptide (WD40) repeats; FBXL, which includes leucine-rich motifs; and FBXO, whose functional domains are less characterized^2^.

The F-box and WD-repeat domain-containing 7 (FBW7) protein plays essential roles in various physiological and pathological cell processes, including facilitating the degradation of various oncoproteins by means of the ubiquitin-proteasome system, thereby regulating cell growth^3^. In many cancers, mutational loss of the substrate recognition subunits in the SCF complex is associated with genomic instability due to the accumulation of its substrates, including but not limited to cyclin E, cyclin D1 and Notch, which contribute to oncogenesis^4^. Moreover, heterozygous mutations in the FBW7 gene are found in multiple cancers^5^. As such, FBW7 is considered a tumor suppressor.

All FBW7 isoforms contain conserved regions including a dimerization (D), F-box and WD40 repeat domains^5^. The WD40 repeat is a short structural motif of ~40 amino acids which often terminates in a tryptophan-aspartic acid (WD) dipeptide. It exhibits a β-propeller architecture and includes multiple four-stranded antiparallel β-sheet blades. As one of the most abundant interacting domains in eukaryotes, WD40-containing proteins function as scaffolds, and thereby enabling protein-protein or protein-DNA/RNA interaction platforms^6^. Due to multiple acidic residues, the WD40 domain of FBW7 presents a negative electrostatic surface charge under physiological conditions. Additionally, it has a hydrophobic pocket that can recognize complementarily charged residues including phosphodegrons in substrate proteins. Studies have shown that the WD40 domain of FBW7 interacts with multiple substrates such as Cyclin E, Sic1 and iso-ADP-ribose based on a complementary electrostatic potential between the binding site of WD40 and the target substrates^7,8^.

Crucial determinants in binding and activation of many proteins are surface charge and flexibility, which have significant impact on signaling molecular interactions involved in cancer biology. Several proteins prominent in cancer biology such as K-ras, c-Src, and Rac1, are affected by high surface charge^9^. Protein flexibility, characteristic of disordered domains lacking a secondary structure, allows multiple protein interactions. These promiscuous protein interactions have been observed in various proteins associated with cancer development including p53, cyclin-dependent kinase (CDK) inhibitors p21^Waf1/Cip1/Sdi1^, p27^Kip1^ and breast cancer type-1 susceptibility protein (BRCA1)^10–13^.

Merkel cell carcinoma (MCC) is an aggressive malignancy with frequent recurrence, high propensity to metastasize and a high mortality rate. MCC is the second most common cause of skin cancer death after melanoma. Although MCC has an incidence rate 30 times lower than cases of malignant melanoma, it is twice as lethal^14^. Merkel cell polyomavirus (MCV) is the causative agent of most MCC^15^. Approximately, 80% of MCC contain clonally integrated copies of MCV DNA, expressing two viral transcripts including a truncated form of large T antigen (LT) and an intact small T antigen (sT)^16^. Although MCV LT has a clear role in promoting tumor cell growth and DNA damage responses^17,18^, MCV sT exerts the dominant oncogenic activity and contributes to tumor development in mouse models^19,20^. A unique domain of MCV sT, the LT stabilization domain (LSD, residues 91–95), has been reported to bind and inhibit FBW7, β-TrCP and CDC20, which leads to enhanced MCV replication, oncogene activation and genomic instability^21–23^. MCV sT is also involved in DNA damage response^24^, cell migration and dissociation^25,26^, and cellular transcriptome/chromatin remodeling;^27,28^ thus, MCV sT is considered the main viral transforming factor required for MCC development. It remains unclear how MCV sT affects this array of disparate activities.

Model prediction of MCV sT revealed a unique large unstructured loop^22^. We hypothesized that the LSD is a disordered and positively charged domain that interacts with a common acidic domain of E3 ligases, WD40, via electrostatic interactions. Herein, we report structural characteristics of the LSD and its interaction with the FBW7 WD40 domain, substantiated by mutational analysis. The effect of WD40 mutations identified in human cancers on its substrate binding was evaluated using FBW7 substrate, MCV LT. Additionally, we further investigated the inhibitory effect of MCV sT on FBW7 E3 ligase. Upon analysis of potential docking models of the sT-FBW7 interaction, a novel model of allosteric inhibition of FBW7 by sT was determined. To alter protein surface charge through traditional protein amino acid engineering approach, we introduced charged amino acid mutations into the LSD. These mutations can modify sT protein properties including its thermostability and functional activity^29^. We infer that modifying the LSD with negative glutamic acid (E) residues ablates the ability of sT to bind FBW7, stabilizes FBW7 substrates, and transforms mouse fibroblast cells as previously observed in alanine (A) mutations^22^. In contrast, modification of the LSD with positive residues lysine (K) and arginine (R) restored sT functions. Together, our data highlight the importance of electrostatics in sT protein-protein interactions that likely plays a role in sT-induced tumorigenesis.

## Results

### A predominant structural mechanism for the sT-E3 ligase interactions

**Fig. 1:**
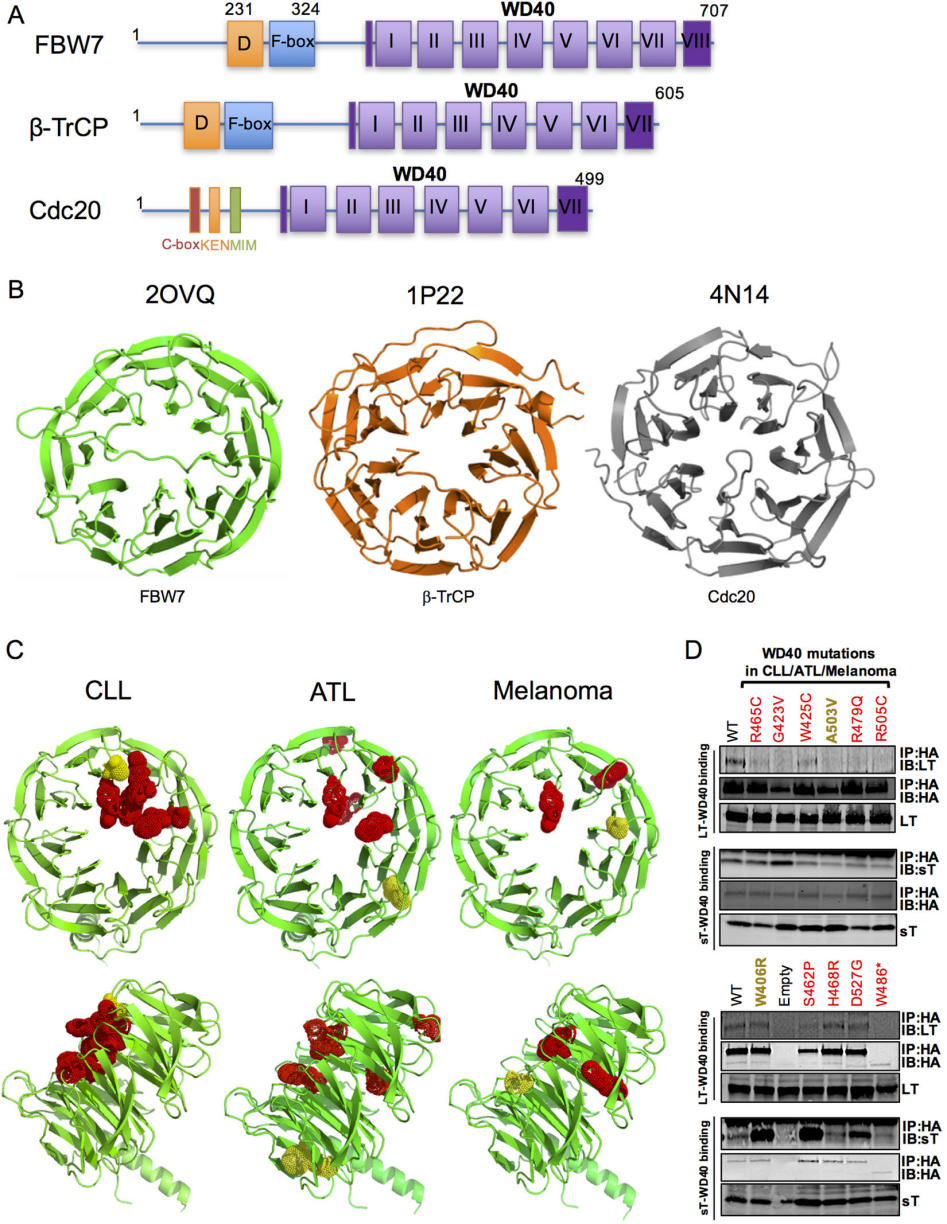
Merkel cell polyomavirus small T targets WD40, a common domain structure of E3 ligases. **a** Schematic organization of FBW7, β-TrCP, and Cdc20 genes. Diagram compares substrate-binding regions of SCF and Anaphase-Promoting Complex/Cyclosome (APC/C): FBW7, β-TrCP, and Cdc20. Color-coded exons corresponding to each specific domain: WD40 repeats mediate substrate recognition (purple); F-box recruits the SCF complex (blue); dimerization domain (orange). Cdc20 has unique domains such as C box, KEN box, Mad2-interacting motif (MIM). A common feature among these E3 ligases is the WD domain. The first β-strand of WD40 completes the last blade structure (dark purple) to create the closed ring propeller-structure. **b** Classical tertiary structure of the WD40 domains of E3 ligases. Ribbon diagrams of WD40 repeat domains of human FBW7, β-TrCP and Cdc20 and their respective PDB ID numbers. **c** Ribbon diagram of FBW7 WD40 domain mutations identified in CLL, ATL and melanoma. β-propeller blades are highlighted in green (top-down and side views). Localization of FBW7 WD40 mutations identified in CLL, ATL and melanoma human cancers are highlighted in red and yellow spheres^30–32^. Mutations contributing to either transforming activity or reducing substrate binding/degradation are depicted as red spheres and mutations without a loss of function are illustrated in yellow spheres. CLL-associated FBW7 mutations are located on a ‘hotspot', the top face of WD40 domain^5^, affecting binding of its substrates to the WD40. In contrast, mutations in ATL and melanoma are less likely to be spatially clustered towards the top face, but instead are buried. **d** WD40 domain mutations in human cancers affect LT, but not sT, binding. Mutations identified in CLL, ATL and melanoma were introduced. The interaction between sT or LT and FBW7 were analyzed by co-immunoprecipitation analysis in 293 cells transfected with LT or sT and HA-tagged WT WD40 (FBW7ΔDF) or mutants. The asterisk (*) indicates a nonsense mutation.

Promiscuous targeting of multiple E3 ligases by sT contributes to its ability to induce genomic instability and cell transformation^21–23^. Notably, these E3 ligases, FBW7, β-TrCP and Cdc20, display a high level of structural homology with each other, sharing a common WD40 domain (Fig. 1a). The WD40 domain forms a β-propeller structure that recognizes and interacts with substrate proteins using the top, the bottom and the circumference of its surface (Fig. 1b)^6^. This implies that sT antigen may prefer to target a predominant structural configuration, such as the WD40 domain, in order to facilitate promiscuous interactions with different WD40 domain-containing E3 ligases. The mechanism by which FBW7 WD40 domain interacts with target proteins is well characterized^5^. Therefore, we focused our study on the potential interaction between MCV sT LSD and FBW7 WD40 domain and the resultant inhibitory effect on FBW7 function.

Although a variety of WD40 proteins and ligand binding modes including allosteric communication are structurally characterized^6,7^, FBW7 substrate interaction studies are relatively limited to those binding residues responsible for charged phosphodegron substrate recognition at the top surface, mostly due to its frequent mutation observed in human cancers. Recently, FBW7 haploinsufficiency and novel mutations in various human cancers have been further explored^30–32^. In these studies, cancer mutations in FBW7 WD40 domain changed substrate recognition due to changes in electrostatic and hydrophobic interactions at the WD40 substrate-binding domain. Thus, we postulate that sT similarly influences WD40 recognition of its substrates, potential proto-oncoproteins in human cancers.

To examine conceptual structure similarities in WD40 inhibition by sT, we performed structural analysis and confirmed the location of FBW7 WD40 cancer mutations identified in chronic lymphocytic leukemia (CLL), adult T-cell leukemia (ATL) and melanoma. Mutations of FBW7 WD40 domain residues that contribute to either transforming activity or reducing substrate NOTCH1 binding/degradation are depicted as red spheres (R465, G423, W425, R479, R505 in CLL; W425, S462, H468, R505, D527 in ATL; G423, R505, W486 in melanoma). Residues without a loss-of-function are illustrated as yellow spheres (A503 in CLL; W406 in ATL; G423 in melanoma) (Fig. 1c). All FBW7 mutations in CLL (R465C, G423V, W425C, A503V, R479Q, R505C) are located in the ‘hotspot’ substrate-binding area, the top face of the WD40 domain. In contrast, certain WD40 mutations associated with ATL (W406R, S462P, H468R, D527G) and melanoma (W486*), are less likely to be spatially clustered towards the top face but are usually found embedded in the β-propeller structure (Fig. 1c). Moreover, loss-of-function mutations (red spheres, Fig. 1c) significantly ablated NOTCH1 binding resulting in NOTCH1 stabilization^30–32^, suggesting the presence of alternative allosteric binding modes for FBW7 NOTCH1 substrate recognition. Considering the allostericity of FBW7 substrates based on this structural analysis, we postulated that sT may abrogate substrate recognition of FBW7 either at the active site of WD40 substrate binding (orthosteric inhibition) or at a site other than the active site (allosteric inhibition) (Fig. S1).

MCV sT prevents FBW7-substrate recognition, stabilizing FBW7 substrates such as c-Myc, cyclin E and LT^22^. To elucidate the mechanism by which sT targets FBW7, we evaluated the interaction between MCV T antigens and FBW7 WD40 cancer mutants by immunoprecipitation analysis. Mutations characterized previously in CLL, ATL and melanoma by dysregulated NOTCH1, a substrate of FBW7, were tested for LT and sT binding^30–32^. For co-immunoprecipitation experiments, we used a FBW7ΔDF construct (WT) to allow expression of wild-type WD40 (deletion of both dimerization and F-box domains that prevents SCF recruitment and thus uncouple substrate binding from its turnover)^33^. Upon introduction of cancer mutations into WT (FBW7ΔDF), the binding of LT to WD40 mutants (R465C, G423V, W425C, A503V, R479Q, R505C and W486*) was diminished (Fig. 1d, Supplementary Table 1). In contrast, ATL-specific WD40 mutations (W406R, S462P, H468R and D527G) did not significantly affect LT interaction. This mutational analysis demonstrated that LT-FBW7 interaction is exclusively affected by mutations on hotspots at the top surface of WD40 domain. However, sT retained its interaction with these WD40 substrate-binding mutants, eliminating the possibility of an orthosteric inhibitory effect. Thus, we concluded that sT may have allosteric inhibitory effects on degradation of FBW7 substrates (Fig. S1). Of note, some of WD40 mutations including G423V, W406R, S462P an D527G showed increased interactions with sT compared to wild-type WD40.

### The allosteric pocket of FBW7-WD40 domain targeted by sT directly engages in substrate interactions

**Fig. 2:**
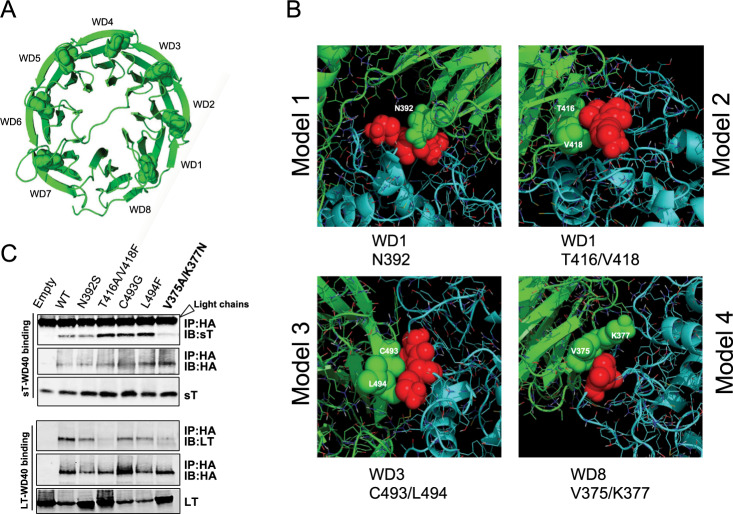
Identification of new allosteric inhibitory sites responsible for sT FBW7 targeting. **a** FBW7 WD40 domain. Ribbon diagram of the 8 WD-repeats in FBW7 WD40. Tryptophan-aspartic acid (WD) dipeptides are depicted as spheres. **b** Inter-residue interactions in MCV sT and WD40 domain docking models. To select and interpret correlated mutations in the WD40, MCV sT (cyan)-WD40 (green) complex structures from 4 potential docking models were analyzed and visualized based on the use of inter-residue contacts (green spears) with amino acids LK (red spears) in LSD. **c** WD8 blade beta propeller is a critical site for both sT and LT interactions. Co-immunoprecipitation assay shows that both MCV LT and sT binding were reduced by FBW7-WD40 mutations in residues V375 and K377 (V375A/K377N). This mutation is located within the WD8 blade.

To understand the structural promiscuity of sT, we utilized a molecular docking approach using ClusPro 2.0 server^34^ to model the interaction between sT and FBW7. ClusPro provided 103 docking configurations as a result of multiple similarities in amino orientation involved in each protein interaction. We selected cut off levels based on the allosteric- and LSD-dependent docked complex in a cluster. A representative cluster of 12 allosteric sT LSD-dependent docking models were analyzed and visualized. Further computational analysis of these 12 allosteric models allowed us to eliminate multiple representations of a specific interaction model, narrowing down our selection to 4 disparate docking models (Fig. S2). For each model, a potential allosteric sT binding region on the β-blades of WD40 repeats (WD1 to WD8) (Fig. 2a) was predicted (Fig. 2b). To confirm a possible allosteric mechanism of inhibition by sT, we selected potential WD40 inter-residue contacts (N392, T416/V418, C493/L494, V375/K377) based on proximity to the LSD surface in these docking models (Fig. 2b and Supplementary Fig. S2). All residues were mutated based on the known amino acid changes observed in the WD40 cancer mutations (N392 to S392, T416/V418 to A416/F418, C493 to G493, L494 to F494 and V375/K377 to A375/N377) and their interaction with either sT or LT was examined by co-immunoprecipitation analysis. When amino acids V375 and K377 in the first β-strand of WD8 repeat were mutated to alanine (A) and asparagine (N), respectively, both LT and sT interactions with this mutant were diminished (Fig. 2c).

**Fig. 3:**
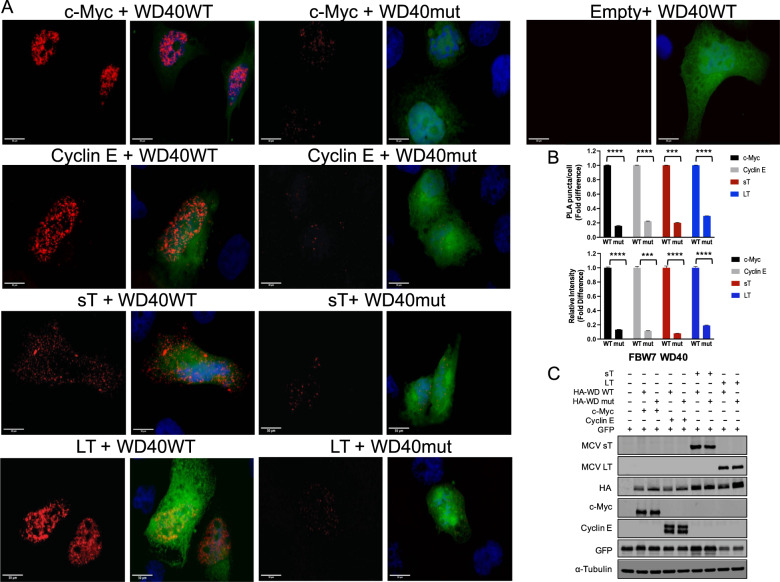
A comparative analysis of FBW7 interaction by proximity ligation assay. **a** MCV LT, c-Myc, Cyclin E and MCV sT were co-expressed with either FBW7ΔDF (WT) or V375A/K377N (mut) in U2OS cells. At 48 h post transfection, a proximity ligation assay (PLA) was performed. Technical controls (Empty vector and FBW7ΔDF (WT)) demonstrate the specificity of PLA signals in samples. Representative images for each sample were prepared by uniformly adjusting brightness and contrast for ImageJ analysis. Red dotted signal indicates interaction and displayed separately. Nuclei were stained with DAPI. GFP plasmid was co-transfected as a transfection control for all samples. GFP fluorescence also indicates the perimeter of the cell. (Scale bar = 30 μm). **b** Quantification of PLA signals. Protein interactions were quantified by counting the number of puncta per cell as well as measuring the florescence intensity of puncta signals per cell. The dots (indicating interactions of PLA probes) per cell were counted by semiautomated image analysis using ImageJ. *n* ≥ 34 cells scored in the experiment. Mean values and standard error of the mean (S.E.M) are represented (***p = 0.0001 for sT (upper panel), ***p = 0.0004 for cyclin E (lower panel), ****p < 0.0001). **c** Protein expression levels of PLA samples. Protein expression was evaluated by immunoblot analysis to validate successful transfection. Quantitative Infrared fluorescence immunoblotting was performed for T antigens, HA-FBW7 (WT and mut), c-Myc, Cyclin E, GFP and alpha-tubulin, respectively.

To evaluate *in situ* protein interactions of FBW7 WD40 domain with a high specificity and sensitivity, we utilized the Proximity Ligation Assay (PLA). Characterized substrates of FBW7 (c-Myc, cyclin E and LT)^22^ and sT antigens were co-expressed with either wild-type (FBW7ΔDF, WT) or mutant form of WD40 construct (V375A/K377N, mut) in U2OS cells to compare the interactions between wild-type and mutant WD40. (Fig. 3a). Quantification of PLA complex puncta number demonstrated that the binding of FBW7 substrates (c-Myc, cyclin E and LT) and sT to V375A/K377N (mut) was decreased as determined from total PLA puncta count in comparison to the wild-type (WT) (Fig. 3b, upper panel). This result was validated by measuring fluorescence intensity in cells that were positive for the interaction (Fig. 3b, lower panel). A reduction in fluorescence intensity was observed when either FBW7 substrates or sT was interacting with mutant WD40 in comparison to WT, consistent with our immunoprecipitation analysis data (Fig. 3c). Additional analysis of the PLA in 293 cells also showed that the PLA frequency was significantly greater in the presence of WT WD40 domain with T antigens (Fig. S3). Although we detected the PLA signal with WD40 mutant (V375A/K377N) co-expression in the overall cell population, the interactions were significantly weaker and displayed fewer puncta and lower fluorescence intensity compared to wild-type (Fig. 3b and Supplementary Fig. S3) under comparable protein expression conditions (Fig. 3c and Supplementary Fig. S3B).

### LSD carries a net positive charge

**Fig. 4:**
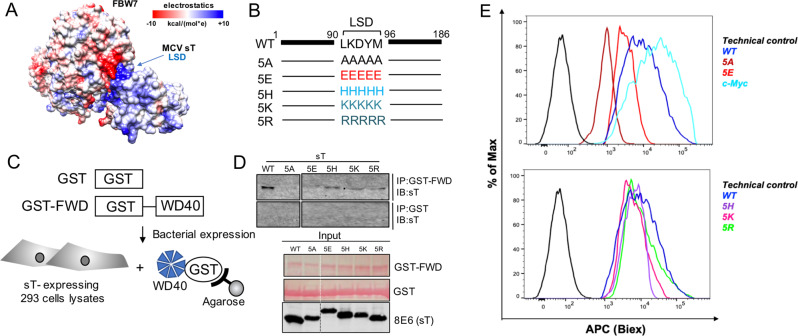
MCV sT LSD is positively charged and required for sT oncogenic function. **a** Electrostatic surface view of MCV sT and WD40 domain structures. The surface electrostatic potentials of WD40 and MCV sT are shown. Positive electrostatic potential is denoted in blue and negative potential in red. LSD residues (91–95) elicit an overall positive electrostatic charge due to the presence of charged amino acids. **b** Mutations in LSD. The LSD amino acids LKDYM (residues 91–95) were mutated to neutral (A), negatively charged (E), weak positively charged (H) and strongly positively charged (K, R) residues to test the effects of surface charge in WD40 interaction and substrate stabilization. **c** GST pulldown assay. The immobilized bait protein (GST or GST-FBW7 WD40 (GST-FWD)) purified from *E*. *coli* was incubated with 293 cell lysates expressing wild-type or mutant sT. **d** Positive charge of LSD is critical for the binding to the WD40 domain. Both neutral (5A) and acidic amino acids (5E) mutations in LSD lost its binding whereas basic amino acids mutation in the LSD restored its binding to the WD40. GST proteins (GST and GST-FWD) were purified and visualized on the membrane using Ponceau S. The bait protein used in each pulldown is >250 ng as detected by Ponceau S (Sigma). 2% of each prey sample was loaded for sT expression detection, using 8E6 antibody. **e** Quantitative analysis of wild-type and LSD mutant sT interactions with the FBW7 using a proximity ligation assay-flow cytometry analysis. A PLA-flow cytometric analysis was performed to further validate the quantitative interaction of FBW7 and LSD mutants. Primary antibodies were utilized at optimized concentrations with HA-Tag (C29F4) rabbit mAb (1:500), c-Myc (9E10) mouse mAb (1:500), and 2T2 (1:500) (Millipore). Protein expression was evaluated by immunoblot analysis in Supplementary Fig. S5C.

Experimental confirmation of sT docking arrangement (model 4) provided the location of the sT LSD-WD40 interface (Fig. 2). Since the WD40 surface is enriched with a continuous positively or negatively charged region on the surface of the β-propeller structure, we analyzed the characteristics of electrostatic potential of the sT-FBW7 complex formation. The electrostatic surface representation of the sT-FBW7 WD40 domain complex highlights a potential electrostatic interaction between the basic region of sT LSD (blue) and acidic residues of WD40 (red) (Fig. 4a and Supplementary Fig. S4). Therefore, to assess whether charge engineering on sT LSD affects sT functions, we constructed a series of LSD mutant derivatives. We altered the overall electrostatic potential of the LSD, changing the original LKDYM sequence to neutral (5 A: AAAAA)^22^, negative (5E: EEEEE), weak positive (5 H: HHHHH) and strong positive (5 K: KKKKK, 5 R: RRRRR) amino acids (Fig. 4b and Supplementary Fig. S5).

Next, we sought to determine if charge engineering of the sT LSD affects its ability to inhibit FBW7 E3 ligase. To evaluate sT and LSD mutant interactions with the WD40 domain of FBW7, in vitro GST pull-down assays were used. The WD40 domain alone was expressed in bacteria and purified. To maintain potential post-translational modifications (PTMs) of sT protein that could influence binding to the complex, all sT wild-type and mutants were expressed in 293 cells (Fig. 4c,d). We observed that mutations in sT LSD altered its protein migration due to changes in electrostatic properties (Fig. 4d). All LSD mutants which retained an overall positive charge (5H, 5K, 5R) partially restored the WD40 interaction compared to wild-type, whereas mutants with both neutral (5A) and negative (5E) charges in the LSD lost the capacity to bind to the WD40 (Fig. 4d). Moreover, we utilized a PLA-flow cytometric analysis to further validate the quantitative interaction of FBW7 and LSD mutants **(**Fig. 4e). Our results validated the GST pull-down data where we observed positively charged LSD mutants (5H, 5K and 5R) retain their interaction with FBW7 WD40 domain while neutral and negative charge mutants (5A and 5E) resulted in significantly reduced interaction.

### Positively charged LSD is required for sT to activate oncoproteins

**Fig. 5:**
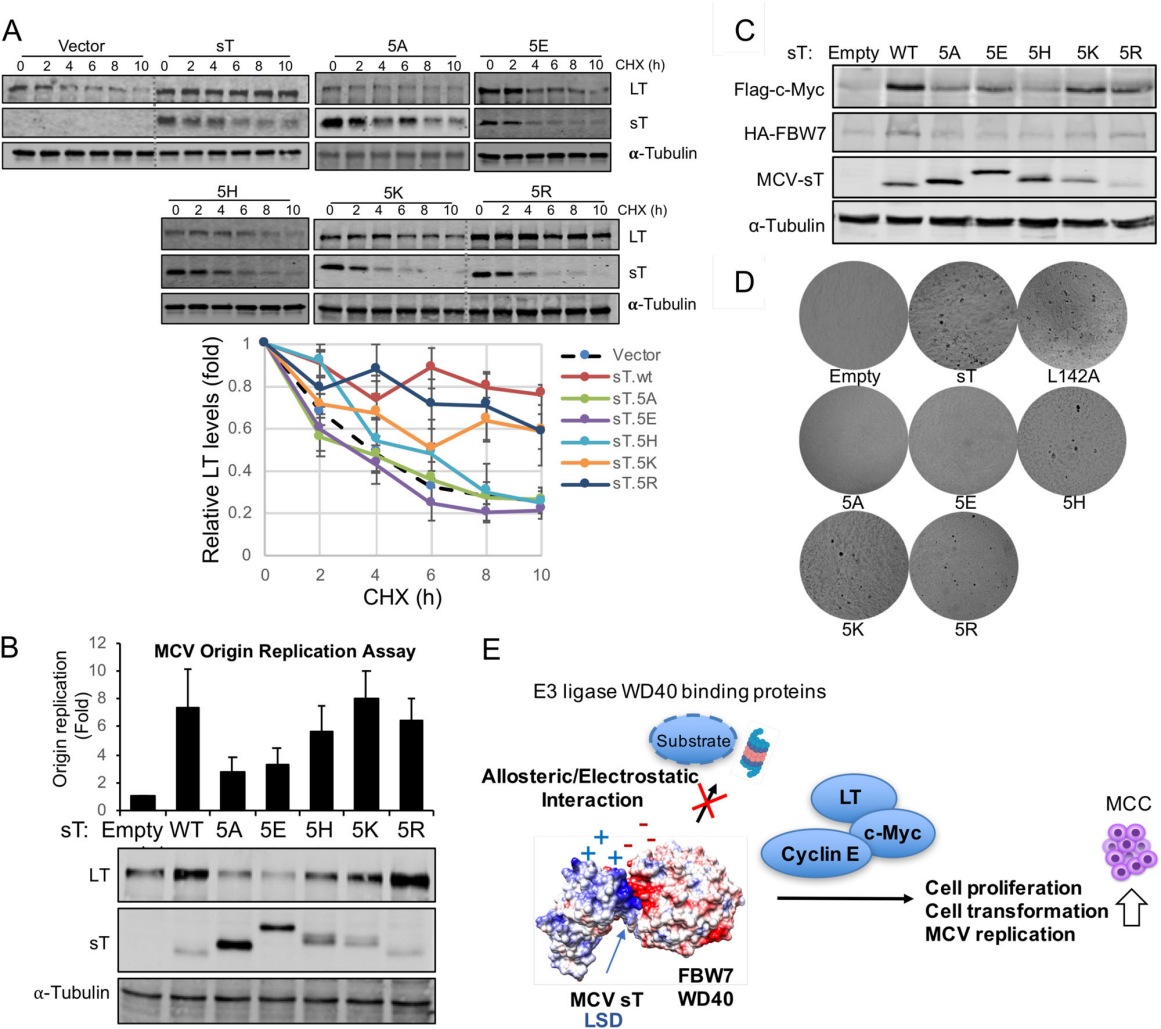
Positively charged LSD surface is required for sT oncogenic function. **a** Positively charged LSD inhibits LT turnover. 293 cells were cotransfected with LT and sT mutant constructs; treated with CHX (0.2 mg/ml) to inhibit new protein synthesis. Cells were harvested at indicated time points. sT mutants with positively charged LSD restored its wild-type function and inhibited LT turnover, with the strongest effect in the 5R mutation. Quantitative immunoblotting was performed for LT, sT and alpha-tubulin detections. Means with error bars representing the S.E.M.; *n* = 3. A representative immunoblot is shown. **b** Positively charged LSD activates LT-mediated MCV origin replication. DpnI-resistant MCV origin replication in 293 cells was assayed by qPCR in the presence of LT alone, or LT together with MCV sT proteins. Error bars represent S.E.M.; *n* = 6. **c** Positively charged LSD activates FBW7 target, c-Myc. 293 cells were transfected with HA-FBW7 WT, Flag-c-Myc and sT constructs. Steady-state levels of c-Myc protein were increased by co-expression of WT sT and positively charged LSD mutants, especially 5K and 5R. **d** Positively charged LSD is critical for sT-induced transformation. Soft agar assay. NIH3T3 cells were stably transduced with vector, wild-type and sT mutants (5A, 5E, 5H, 5K, 5R, and L142A). Wild-type sT, L142A mutants and basic LSD mutants (5H, 5K, 5R) reproducibly formed colonies after 5 weeks of growth in soft agar, whereas the sT 5A and 5E mutations ablated transforming activity. Transformed foci on the surface of soft agar were photographed (×40). All assays were performed in triplicates (Supplementary Fig. S6). PP2A binding mutant, L142A, was used as positive control for transformation. **e** Molecular mechanisms of oncogene activation by MCV sT. The LSD inhibits E3 ubiquitin ligase activity via allosteric and electrostatic surface interactions with the WD40 domain, resulting in increased oncoprotein stability to activate viral replication and cell proliferation.

To evaluate the turnover of the FBW7 target protein as a result of LSD charge engineering, we assessed LT protein accumulation by MCV sT upon mutation of charged residues in LSD. Previous study has shown that in the absence of sT, LT had a rapid turnover with a half-life (t_1/2_) of ∼4 h and diminished to low levels within 10 h after cycloheximide (CHX) addition^22,35^. LT protein turnover was measured by a cycloheximide (CHX) chase assay using a quantitative immunoblot analysis. Co-expression of sT and LT increased the t_1/2_ for LT to >10 h, as previously shown (Fig. 5a)^22^. The effect was lost in the presence of either sT mutants 5A or 5E. In contrast, the co-expression of positively charged LSD mutants (5K and 5R) led to accumulation of LT protein after 10 h of CHX treatment (Fig. 5a). It should be noted that the 5H (Histidine pK = ~6) mutations had a minimal effect on inhibiting LT turnover (*t*_1/2_ = ~6 h), while the LSD mutants with highly positively charged arginine (pK = 12.5) and lysine (pK = 10.5) almost completely restored its function compared to wild-type sT, effectively diminishing LT degradation (*t*_1/2_ > 10 h). This suggests that the positively charged residues in the LSD of sT act to stabilize LT protein.

MCV sT expression activates MCV origin replication and MCV genome replication due to increased stability of LT protein^22^. Using an MCV origin replication assay, we tested various sT LSD constructs for their effects on MCV LT-dependent replication activity. MCV origin replication was greatly activated by sT and LSD mutants with the positive charged residues (5K and 5R) (Fig. 5b), whereas sT mutant 5H had a minimal effect. This effect was nearly ablated with sT bearing either alanine (5A) or glutamic acid (5E) substitutions in the LSD. Consistent with sT targeting FBW7 to enhance steady-state LT expression and viral replication, sT and positive LSD mutants increased cellular oncoprotein, c-Myc (Fig. 5c).

### Positively charged LSD determines sT transforming activity

Mutation of the LSD not only abolished MCV sT stabilization of MCV LT but also eliminated sT-induced rodent cell transformation function^19,22^. To determine the effect of LSD charge on sT-induced cell transformation, we performed soft agar colony growth assays. Lentiviral transduction was performed in NIH3T3 mouse fibroblast cells with wild-type sT and the various sT mutants. A PP2A binding mutant of sT, L142A, which transforms cells through a PP2A-independent mechanism served as a positive control^36,37^. Similar to wild-type MCV sT, positively charged LSD mutants (5H, 5K, 5R), reproducibly formed colonies after 5 weeks of growth in soft agar (Fig. 5d and Supplementary Fig. S6A). This alteration in colony formation occurred in the setting of comparable levels of sT expression (wild-type and sT mutants) (Supplementary Fig. S6B). In contrast, the MCV sT 5 A and 5E mutations ablated transforming activity of sT. Of note, 5K and 5R mutants have shown significantly lower expression in comparison to sT wild-type and other mutants (Fig. 5b,c). However, low levels of 5K and 5R were sufficient to inhibit FBW7 degradation of MCV LT and c-Myc, supporting MCV replication and cell transformation.

## Discussion

Surface charge has emerged as a significant determinant of global cellular functions in multiple tumor suppressors and oncoproteins^38,39^. Our study identified a previously undescribed charged allosteric interface in the FBW7 WD40 domain that is responsible for interacting with FBW7 substrates such as MCV LT, c-Myc and cyclin E, highlighting the complexity that has evolved within the WD40 protein family interactions. Mutations in allosteric pockets potentially lead to structural changes, resulting in change of local hydrophobicity or electrostatic potentials that is critical for substrate-binding to the WD40 domain of FBW7^31^. Although a specific mechanism is unknown, small changes in the position of charged residues in the allosteric pocket may also induce primary structural changes, leading to a loss of substrate interaction^40^. It is expected that both sT and substrate interactions would be abolished by mutating this allosteric pocket, if this mutation alters its structural conformation or charge-state distribution of substrate-binding region. It is not clear whether this allosteric interface is involved in direct contact of substrates. Nonetheless, our results demonstrate that various binding modes of WD40-protein substrate interactions exist.

The possibility of dynamic substrate recognition and multiple binding pockets in an E3 ligase was recently shown in a study that confirmed the discovery of a secondary allosteric pocket in SCF^Cdc4^, which in turn alters substrate recognition^7^. Although FBW7 substrates are mainly known to interact at the top face of the WD40 domain, it is possible that each WD40 protein may have a unique way to process its substrate degradation, as implicated in other cancer mutation studies^30–32^. Specifically, identification of E3 ligase substrate by immunoprecipitation is challenging due to the weak physical interaction and rapid dissociation rate between some E3–substrate complexes^41^. This dynamic equilibrium between E3 ligases and substrates highlights the need for more sensitive techniques such as PLA. Although our initial interaction studies performed by immunoprecipitation may present some technical variations, yet were able to display a glimpse into the perspectives of these interactions.

Given that sT does not have the canonical Cdc4 phosphodegron (CPD) motif, it would not be logical to assume that sT competes for substrate binding to the FBW7 WD40 at the same location. Our analysis of sT and FBW7 WD40 domain docking structure provided a major surprise, revealing that sT acts as an allosteric inhibitor rather than a direct competitive inhibitor, similar to SCF-I2 allosteric inhibitor of Cdc4 WD40^7^. Additionally, we expanded our mutational spectrum of the LSD to define the significance of surface charge in sT-E3 ligase complexes, which we validated with functional assays that can provide physiological information beyond the physicochemical patterns of protein interactions. An important question that remains to be addressed is how sT LSD presents a positive charge. Post-translational modification of proteins is important for the regulation of cellular processes including protein localization, regulation of protein function and protein complex formation. None of the post-translational protein modifications on sT have been extensively characterized. The sT LSD contains lysine (K), which is mostly exposed to the protein surface, and may play a major role in sT-induced oncoprotein stabilization by forming electrostatic and/or hydrophobic interactions with E3 ligases (Supplementary Fig. S4). The LSD amino acid composition (LKDYM) is similar to conserved monomethyl lysine sites identified in human cancer cells^42^, suggesting a possibility of potential lysine modification in the LSD. Therefore, future studies of potential protein modifications in the LSD will need to be implemented.

The disordered and charged surface properties of the LSD are complementary characteristics that mediate sT-E3 ligase interactions. The LSD is conserved across all MCV isolates and maintains a partial structure within the large and disordered loop. The mutagenesis of this disordered region may cause a change in composition and the distribution of intrinsic charge density and hydrophobicity. Therefore, is not surprising that other MCV sT-specific domains (EP400 binding site and PP4C binding site), located within this unique unstructured loop are also important for other sT interactions and functions^26,28^. Nonetheless, it is clear that the unique disordered segment contributes to sT-mediated oncogenicity. It also should be noted that changes in hydrophobic and/or electrostatic interactions between the FBW7 WD40 and sT may result in altered structural stability of WD40 and recognition of yet unknown substrates due to sT expression. These changes may lead to modified binding affinity as a result of altered conformational entropy caused by molecular dynamics of protein complexes.

To our knowledge, MCV sT is the first allosteric viral protein inhibitor that targets WD40 proteins and sT targeting of WD40 protein family may facilitate dynamic exchange of multiple substrate interactions and protein proteolysis processes in MCC. Undoubtedly, structural properties are not the only factors that play a defining role in the ability of proteins to interact with multiple partners. Other important properties such as the localization, homeostasis of cellular proteins in the cell, and the binding affinities of the various targets will also have a large impact on the number of proteins that may interact with sT. Further structural studies will be required to provide a more refined view of sT oncoprotein and its targeting of WD40-containing cellular proteins in MCC oncogenesis. Additionally, these WD40-containing E3 ligases are known to limit MCV replication and reactivation^35^. Although a coding polymorphism of the WD40 in MCC has not been explored, a polymorphic dysregulation of E3 ligases in MCC may have significant impacts on activation of MCV replication and reactivation by sT that might be important for oncogenic potential of MCV.

In summary, this study reports a novel interacting mechanism of cellular tumor suppressor FBW7 with the viral oncoprotein MCV sT (Fig. 5e) and characterizes its functional impact in MCC as a relevant therapeutic target. Deciphering cellular pathways regulated by sT LSD may lead to novel anticancer therapeutic interventions for MCC patients who currently lack therapies.

## Materials and methods

See “Supplementary Materials and methods” section for additional details.

### Plasmids and cell lines

Primer sequences for the constructions and plasmids used for this study were listed in Supplementary Tables 2 and 3, respectively. U2OS and 293 cells were cultured in DMEM with 10% premium grade fetal bovine serum (FBS) (Seradigm). NIH3T3 cells were maintained in Dulbecco’s Modified Eagle Medium (DMEM) with 10% Bovine calf serum (Seradigm).

### MCV origin replication by quantitative PCR analysis

The MCV replication origin assay has been previous described^43^. 293 cells were transfected with expression vector (LT/sT) and pMCV-Ori339(97) using Lipofectamine 3000 (Invitrogen) in 12-well plates. Episomal DNA was collected and subjected to qPCR.

### Structural analysis and docking modeling

PDB structures of 2OVQ, 1P22, 4N14 were used^44–46^ for WD40 domain analysis (Fig. 1). The model of MCV sT structure was generated using the I-TASSER server^47^ based on SV40 sT homolog structures (PDB ID: 2PF4, 2PKG)^22^. The ClusPro server (https://cluspro.org) is used a tool for sT-FBW7 docking modeling^34^.

### Co-immunoprecipitation (co-IP) assays

Cells were lysed in IP buffer (50 mM Tris-HCl (pH 7.4), 150 mM NaCl, 1% Triton X-100) freshly supplemented with 1 mM phenylmethylsulfonyl fluoride (PMSF), and 1 mM benzamidine. Lysates were incubated at 4 °C overnight with 20 μl 50% slurry of anti-HA Agarose beads (Pierce) completely equilibrated with IP buffer. Beads were washed with IP buffer and high salt IP washing buffer (50 mM Tris-HCl (pH 7.4), 500 mM LiCl). Beads were resuspended in 2×SDS loading buffer, and all proteins were separated by SDS-PAGE (4–20% Criterion^TM^ TGX^TM^ precast gradient protein gels) followed by immunoblotting to detect interacting proteins.

### Immunoblotting and Antibodies

Cells were lysed in IP buffer and sonicated whole cell lysates were used for direct immunoblotting. Primary antibodies were incubated overnight at 4 °C, followed by 1 h secondary antibody incubation at RT. All signals were detected using quantitative Infrared (IR) secondary antibodies (LI-COR). Signal intensities were analyzed using a laser-scanning imaging system, Odyssey CLX (LI-COR). Antibodies used for this study were listed in Supplementary Table 4.

### Soft agar assay

NIH3T3 cells stably expressing sT antigens were seeded over a 0.6% agar layer in a 6-well plate (2.5×10^4^ cells/well) and grown for 5 weeks. Cells were selected with puromycin (3 µg/ml) after infection followed by transient transduction with pLVX empty vector, wild-type sT (sT.wt) and LSD mutants, and subjected to transformation assays. Phase-contrast images of cells in soft agar were taken using an Olympus CKX31 inverted microscope (×40).

### GST pulldown assay

Two days after transfection, 293 cells were lysed with immunoprecipitation lysis buffer and pre-cleared. GST-FWD plasmid was transformed into *E. coli* Rosetta(DE3)pLysS strain (Novagen) for FBW7 WD40 domain expression. The WD40 protein was purified using glutathione-Sepharose 4B (GE Healthcare, Cat#17–0756–01) according to the manufacturer’s protocol.

### Proximity ligation assay (PLA) and Flow cytometry

PLA was performed using Duolink In Situ Detection Reagents or flowPLA Detection kit (Sigma-Aldrich) according to the manufacturer’s instructions. Fluorescence micrographs were collected by a REVOLVE4 fluorescent microscope (Echo Laboratories). For PLA flow cytometry, cells were analyzed by flow cytometry on a 16-color BD LSR Fortessa. The acquired data were analyzed using FlowJo software (Tree Star, Ashland, OR, USA).

## Supplementary information

Supplemental material
