# Supplementary material for: Surface charge of Merkel cell polyomavirus small T antigen determines cell transformation through allosteric FBW7 WD40 domain targeting
Source: Oncogenesis. 2020 May 19;9(5):53. doi: 10.1038/s41389-020-0235-y (PMC7237485; doi:10.1038/s41389-020-0235-y)
Supplement: Supplementary file 1 — Supplemental material [file 41389_2020_235_MOESM1_ESM.pdf]

Supplementary Information for

Surface charge of Merkel cell polyomavirus small T antigen determines cell transformation through allosteric FBW7 WD40 domain targeting

Nnenna Nwogu[1], Luz E. Ortiz[1], Hyun Jin Kwun[1,*]

[1]Department of Microbiology and Immunology, Pennsylvania State University College of Medicine, Hershey, PA, USA

*Corresponding author: Hyun Jin Kwun
Email: hxk479@psu.edu

This PDF file includes:

Figures S1 to S6
Tables S1 to S4
Supplementary Materials and Methods
References

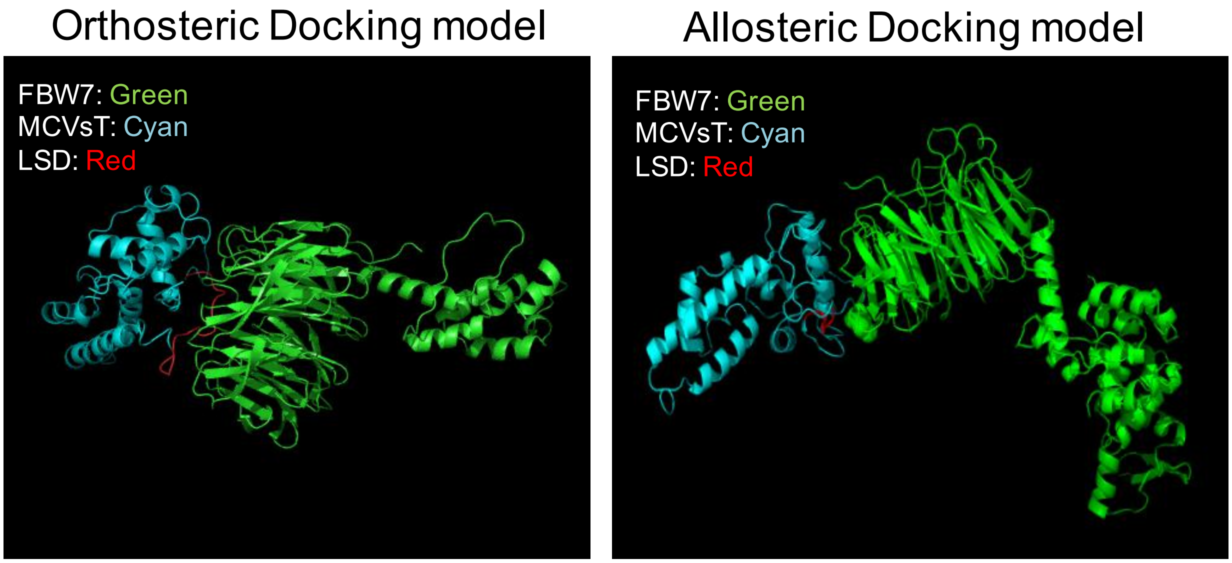

Fig. S1. Potential docking models of MCV sT and FBW7 E3 ligase. The WD40 domain of FBW7 has a hydrophobic pocket that recognizes negatively charged phosphodegrons in substrate proteins. The majority of substrates targeted for degradation are recognized by the top surface of the β-propeller architecture (**Fig. 1**), which is typically mutated in many human cancers. Alternatively, allosteric inhibitors and mutations located on the sides of the structure, specifically the blades, force the WD40 propeller to an open conformation allowing substrate perturbation potential. MCV sT (cyan) is known to inhibit FBW7 (Green) substrate degradation through the LSD domain (red). Two potential modes of inhibition by sT are predicted by molecular docking approaches. Structural prediction for inhibition of FBW7 substrate recognition by sT is illustrated in the figure above. MCV sT may directly interact and inhibit orthosteric substrate binding or allosterically interact and force to change the WD40 conformation, allowing a disturbance of substrate interaction with the WD40 domain of FBW7.

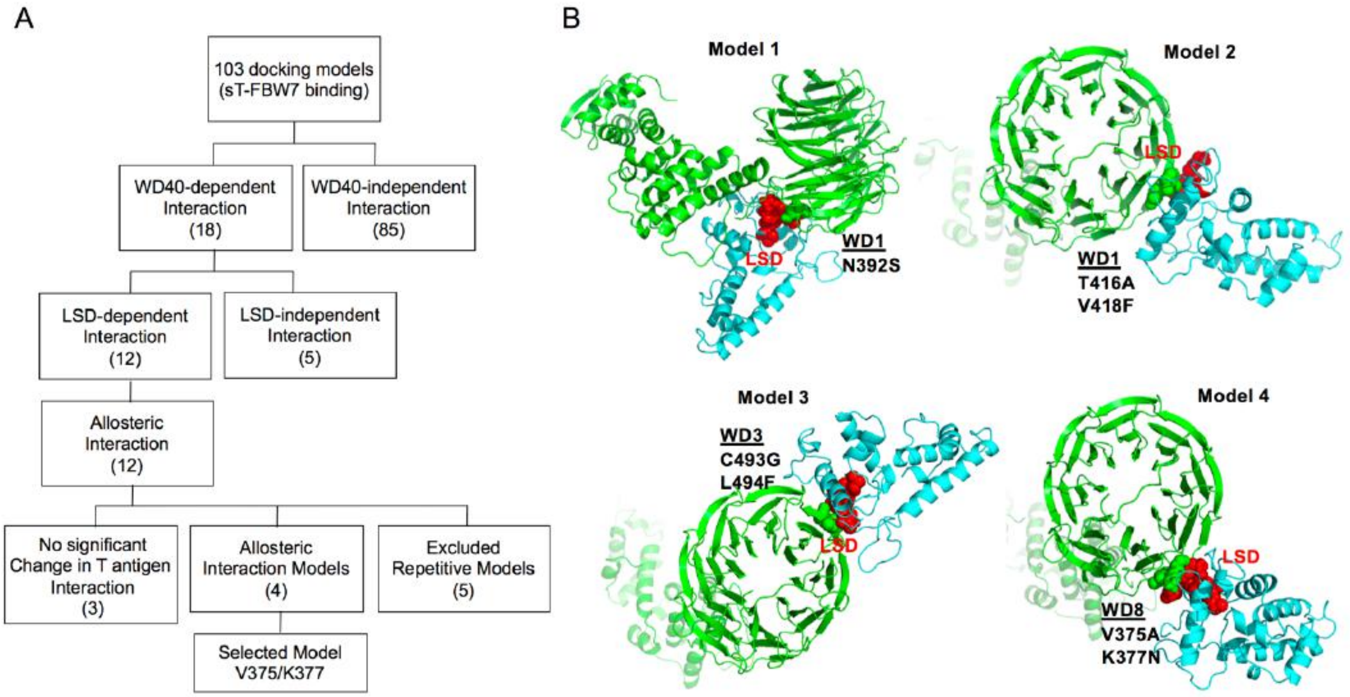

Fig. S2. Potential allosteric sT docking models. To confirm a possible inhibitory mechanism of inhibition by sT, 103 docking models of sT and FBW7 were analyzed. **(A)** Shown here is a flow chart summarizing the workflow of docking model analysis. 103 docking models were initially investigated. 85 models where excluded as the interactions were not WD40-dependent. Another 5 LSD-independent models were excluded. 5 more models where disqualified as they were repetitive while another 3 was did not make the cut due to no significant alteration in T antigen binding. Four potential allosteric LSD-dependent sT docking models were selected and further tested. **(B)** Four potential allosteric LSD-dependent sT docking models summarized. Potential binding residues (N392, T416/V418, C493/L494, V375/K377) were selected for mutation by visual examination of the residual potential (**Fig. 2**).

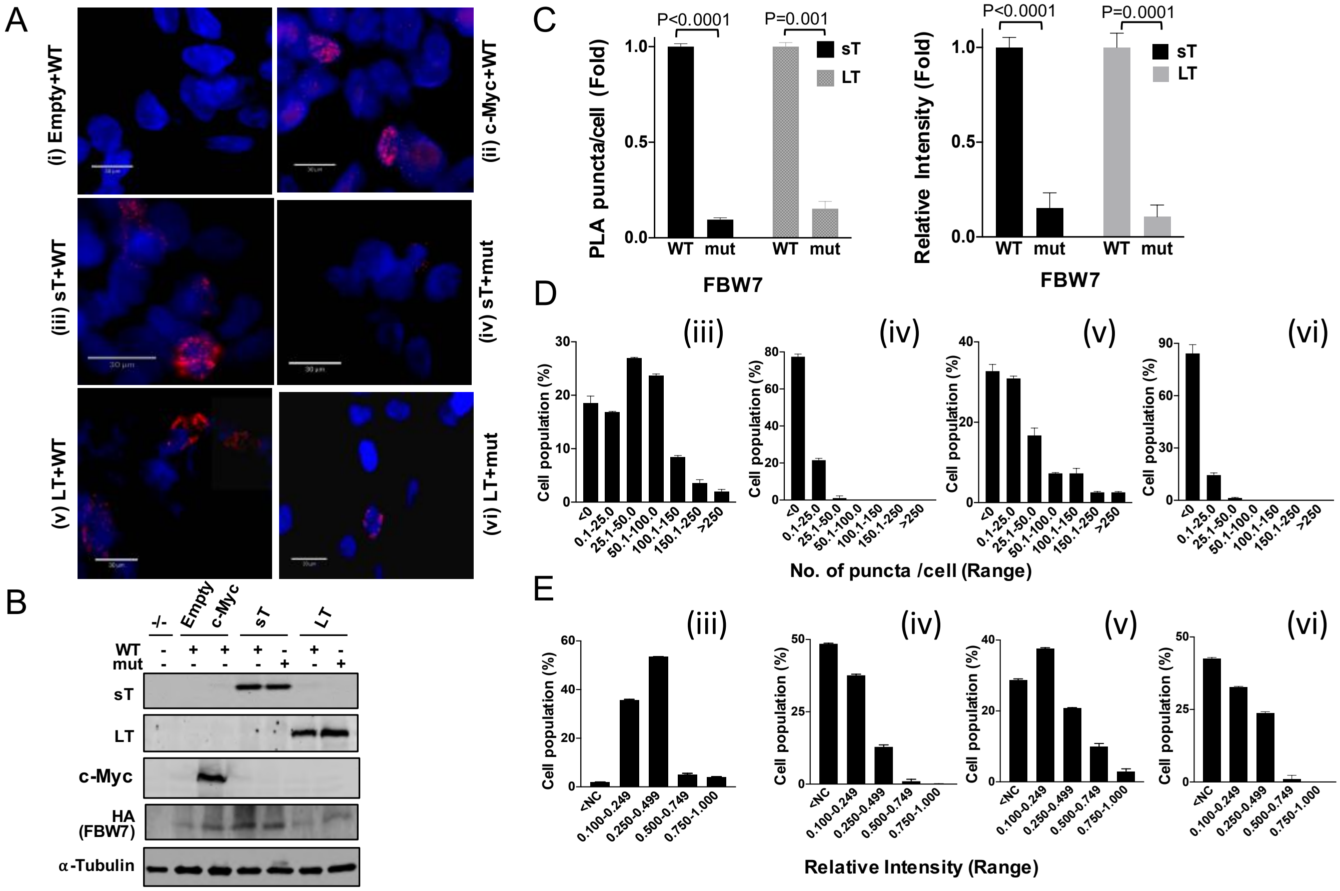

Fig. S3. A comparative analysis of sT and LT interaction with FBW7. (A) Proximity ligation assay (PLA) validates the interaction of sT and LT with FBW7 WD40. Fluorescence microscopy, from left to right: PLA reaction in 293 cells transfected with (i) Empty vector and FBW7ΔDF (WT), (ii) c-Myc and FBW7ΔDF (WT), (iii) sT and FBW7ΔDF (WT), (iv) sT and V375A/K377N (mut), (v) LT and FBW7ΔDF (WT), and (vi) LT and V375A/K377N (mut). Technical controls ((i) and (ii)) demonstrate the specificity of PLA signals in samples with both LT and sT and with wild-type (WT) FBW7 WD40. Representative images for each sample were prepared for presentation by uniformly adjusting brightness and contrast for ImageJ analysis. **(B)** Protein expression levels of PLA samples. Protein expression ((i)-(vi)) was detected by immunoblot analysis to validate successful transfection. Quantitative Infrared fluorescence immunoblotting was performed using 2T2, 9E10, C29F4, 12G10 antibodies for T antigens, c-Myc, HA-FBW7 (WT and mut) and alpha-tubulin, respectively. **(C)** Protein interactions were quantified by counting the number of puncta per cell (left) as well as the intensity of signal per puncta (right). Plot of percentage of fluorescence intensity, normalized to non-fluorescent cells. The dots (indicating interactions of PLA probes) per cell were counted by semiautomated image analysis using ImageJ. n ≥ 250 cells scored in the experiment. Mean values and standard errors are represented. d, e, Counts and intensity distribution of PLA. Additionally, the distribution of count **(D)** and fluorescence intensity of signal per dot **(E)** for the PLA positive cells are also represented.

Electrostatic surface charge of sT LSD

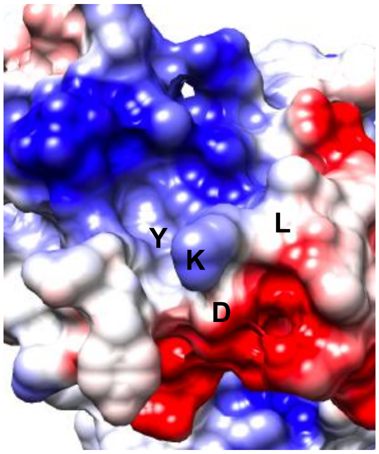

Fig. S4. Electrostatic surface view of MCV sT LSD. The surface electrostatic potentials of MCV sT LSD is shown where positive electrostatic potential is denoted in blue and negative potential in red. LSD has a feature surrounded with charged amino acids. In our model, the strong negative electrostatic field in WD40 interacts with the positive electrostatic field of sT LSD. While methionine (M) is buried inside the pocket, lysine (K) in LSD (LKDYM) is mostly exposed to protein surface, and may play important roles in sT stability and targeting cellular proteins by forming electrostatic interactions.

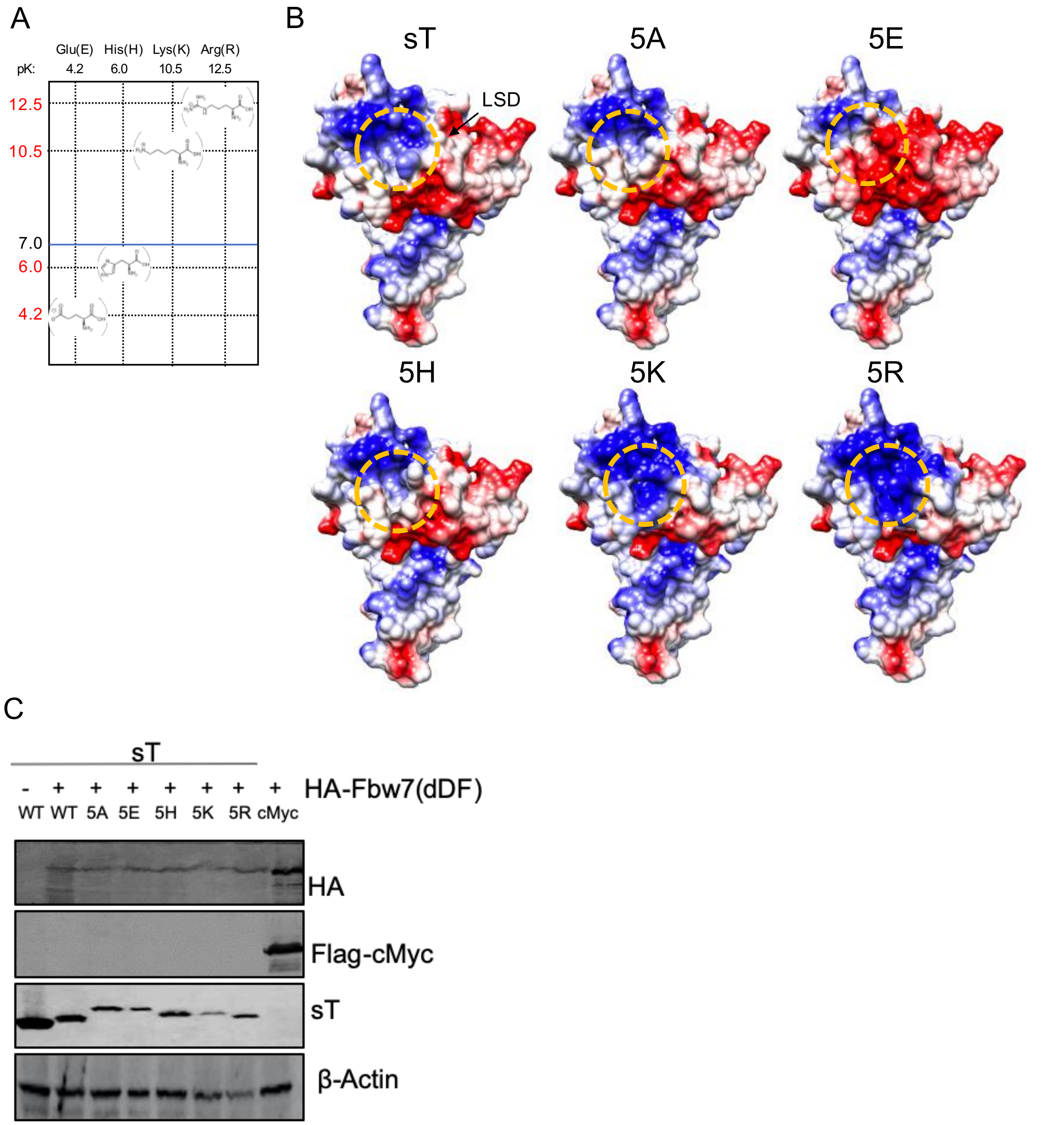

Fig. S5. Charge engineering of LSD. (A) pK of the amino acid side chain groups used in this study. Histidine has a pK of approximately 6.0, close to physiological pH. At a pH inferior to their pK, the lysine (pK=10.5) and arginine (pK=12.5) are highly basic. **(B)** Electrostatic surface view of MCV sT and sT mutants used in this study. An important characteristic of the mutation output on the LSD surface are provided between the wild type sT and mutants (5A, 5E, 5H, 5K, and 5R). LSD mutations were generated via the rotamers function showing different rotameric states using Chimera10. Selected positioning was based on their highest probability and proximity to other residues. Electrostatic coloring of structural surfaces was based on the electrostatic potential via the Coulombic preset. **(C)** Protein expression levels of PLA for flow cytometry (**Fig. 4E**). Protein expression was evaluated by immunoblot analysis to validate successful transfection. Quantitative Infrared fluorescence immunoblotting was performed for sT antigen, HA-FBW7, Flag-c-Myc, and β-Actin respectively.

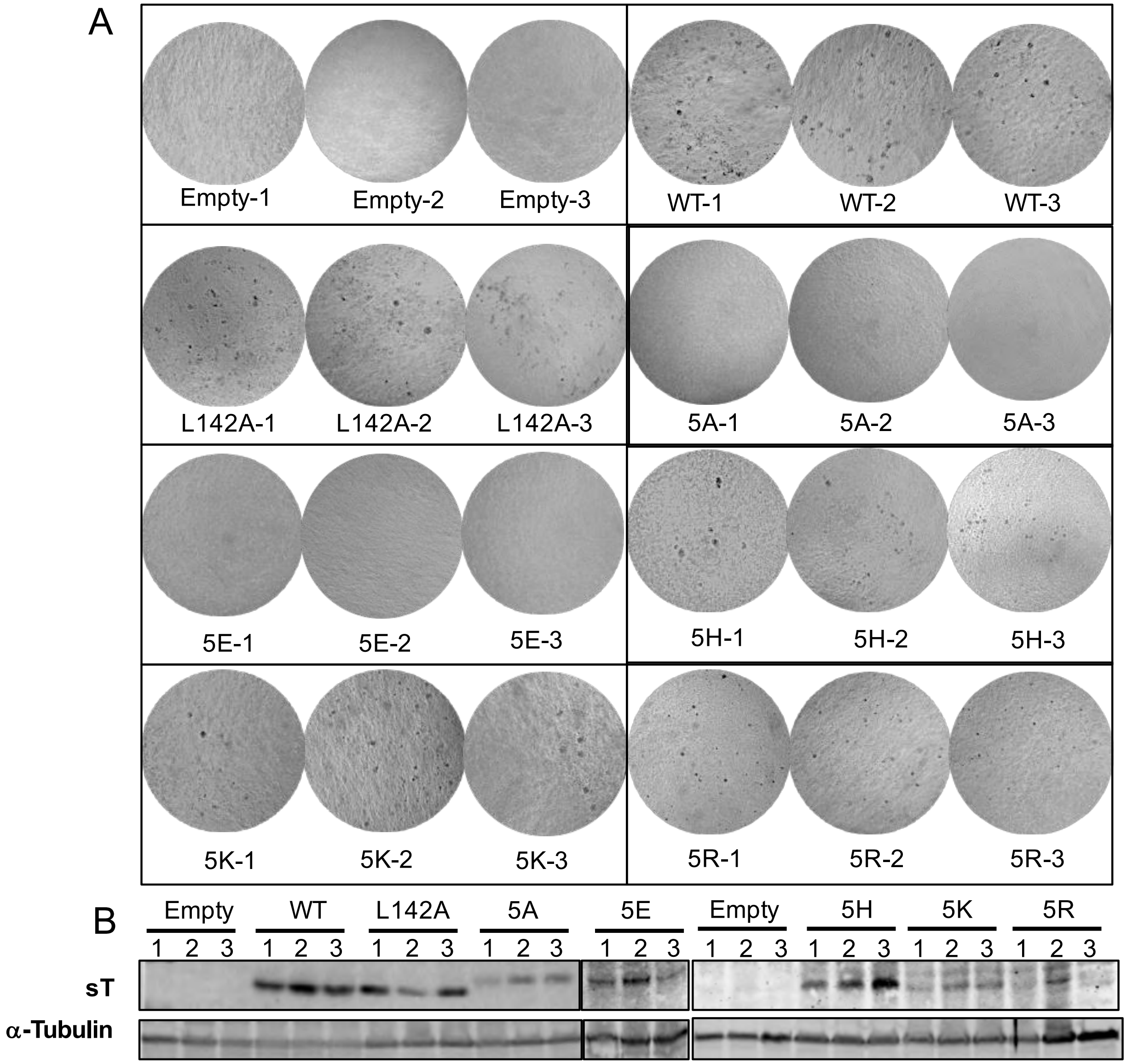

Fig. S6. Positive charge of LSD is required for sT-induced cell transformation. (A) Soft agar assay. NIH3T3 cells were stably transduced with vector, wild-type and sT mutants (5A, 5E, 5H, 5K, 5R, and L142A). Both wild-type MCV sT and L142A mutants reproducibly formed colonies after 5 weeks of growth in soft agar, whereas the MCV sT 5A and 5E mutations ablated transforming activity. Similar to wild-type MCV sT, basic LSD mutants (5H, 5K, 5R), reproducibly formed colonies. Transformed foci on the surface of soft agar were photographed (×40). All assays were performed in triplicates. **(B) sT expression.** Colony formation occurred in the setting of comparable levels of sT expression (WT and sT mutants).

Table S1. Summary of mutational analysis.

FBW7 mutation	Associated Cancer(s)	Change in phenotype	Location	MCV LT binding	MCV sT binding
R465C	CLL	Reduced substrate binding [1]	Hotspot at the top face	-	+
G423V	CLL, Melanoma (G423R)	Reduced substrate binding [1], no effect on tumor growth [2]	Hotspot at the top face	-	++
W425C	CLL, ATL (W425R)	Reduced substrate binding [1, 3]	Hotspot at the top face	-	+
R479Q	CLL	Reduced substrates recognition [1]	Hotspot at the top face	-	+
R505C	CLL, ATL, Melanoma	Reduced substrate recognition [1, 3], increased tumor growth [2]	Hotspot at the top face	-	+
S462P	ATL	Reduced substrate recognition [3]	β-propeller	+	++
H468R	ATL	Reduced substrate recognition [3]	β-propeller	+	+
D527G	ATL	Reduced substrate recognition [3]	β-propeller	+	++
W486*	Melanoma	Increased tumor growth [2]	β-propeller	-	+
A503V	CLL	No loss of function [1]	Hotspot at the top face	-	+
W406R	ATL	Reduced substrate recognition [3]	β-propeller	+	++

* (asterisk) = translation termination (stop) codon.
Mutations in red (R465, R479, R505): the most frequent hot spot mutations occurred in human cancers [4].

Table S2. Primers used in this study.

Name	Sequence (5' to 3')	Notes
sT.5K.F	GGCACAaagaagaagaagaagCAGAGTGGCTAC	sT.5K
sT.5K.R	GCCACTCTGcttcttcttcttcttTGTGCCATATTCC	sT.5K
sT.5H.F	AATATGGCACAcaccaccaccaccacCAGAGTGGCTAC	sT.5H
sT.5H.R	GCCACTCTGgtggtggtggtggtgTGTGCCATATTCCTC	sT.5H
sT.5E.F	GGAATATGGCACAgaagaggaggaggagCAGAGTGGCTACAATGC	sT.5E
sT.5E.R	GTAGCCACTCTGctcctcctcctcttcTGTGCCATATTCCTCCC	sT.5E
sT.5R.F	GAATATGGCACAcgcaggaggcgcaggCAGAGTGGCTAC	sT.5R
sT.5R.R	GTAGCCACTCTGcctgcgcctcctgcgTGTGCCATATTCCTC	sT.5R
AfeI_ST to pLVX_F	gcaagcAGCGCTaccATGGACTTGGTCC	pLVX-sT clones
BHI_ST to pLVX_R	cctGGATCCTCAGAAGAGATGCAAG	pLVX-sT clones
b-globin intron.R	CAACACCCTGAAAACTTTGCCCCC	Fbw7, WD domain reverse primer
Fbw7.R465C.F	CATACTTCCACTGTGTGTTGTATGCATCTTCATG	Fbw7 WD domain mutation
Fbw7R465C.R	CATGAAGATGCATACAACACACAGTGGAAGTATG	Fbw7 WD domain mutation
WD40-G423V.F	gggacatacaggtGTAgtatggtcatcac	Fbw7 WD domain mutation
WD40-G423V.R	gtgatgaccatacTACacctgtatgtccc	Fbw7 WD domain mutation
WD40-W425C.F	catacaggtggagtaTGTtcatcacaaatgag	Fbw7 WD domain mutation
WD40-W425C.F	ctcatttgtgatgaACAtactccacctgtatg	Fbw7 WD domain mutation
WD40-A503V.F	ggtcatgttgcaGTAgtccgctgtgttcaat	Fbw7 WD domain mutation
WD40-A503V.R	attgaacacagcggacTACtgcaacatgacc	Fbw7 WD domain mutation
R479Q.F	gttgttagcggttctCAAgatgccactcttag	Fbw7 WD domain mutation
R479Q.R	ctaagagtggcatcTTGagaaccgctaacaac	Fbw7 WD domain mutation
R505C.F	catgttgcagcagtcTGCtgtgttcaatatg	Fbw7 WD domain mutation
R505C.R	catattgaacacaGCAgactgctgcaacatg	Fbw7 WD domain mutation
W406R.F	caacactttaaaagttCGGtcagcagtcacaggc	Fbw7 WD domain mutation
W406R.R	gcctgtgactgctgaCCGaacttttaaagtgttg	Fbw7 WD domain mutation
S462P.F	cttatatgggcatactCCCactgtgcgttgtatg	Fbw7 WD domain mutation
S462P.R	catacaacgcacagtGGGagtatgcccatataag	Fbw7 WD domain mutation
H468R.F	ccactgtgcgttgtatgCGTcttcatgaaaaaagag	Fbw7 WD domain mutation
H468R.R	ctcttttttcatgaagACGcatacaacgcacagtgg	Fbw7 WD domain mutation
D527G.F	attttatggtaaaggtgtggGGTccagagactgaaacc	Fbw7 WD domain mutation
D527G.R	ggtttcagtctctggACCccacacctttaccataaaat	Fbw7 WD domain mutation
W486tga.F	gccactcttagggttTGAgatattgagacag	Fbw7 WD domain mutation
W486tga.R	ctgtctcaatatcTCAaaccctaagagtggc	Fbw7 WD domain mutation
N392S-F	cttacagttttgtggtAGCcgaatagttagtggttc	Fbw7 WD domain mutation
N392S-R	gaaccactaactattcgGCTaccacaaaactgtaag	Fbw7 WD domain mutation
T416V418AF-F	ggcaaatgtctgagaGCAttaTTCggacatacaggtggag	Fbw7 WD domain mutation
T416V418AF-R	ctccacctgtatgtccGAAtaaTGCtctcagacatttgcc	Fbw7 WD domain mutation
C493G.F	gatattgagacaggccagggtttacatgttttgatg	Fbw7 WD domain mutation
C493G.R	catcaaaacatgtaaaccctggcctgtctcaatatc	Fbw7 WD domain mutation
L494F.F	gagacaggccagtgtTTTcatgttttgatggg	Fbw7 WD domain mutation
L494F.R	cccatcaaaacatgAAAacactggcctgtctc	Fbw7 WD domain mutation
V375K377AN-F	ctcaaatctcctaagGCGctgAATggacatgatgatcatg	Fbw7 WD domain mutation
V375K377AN-R	catgatcatcatgtccATTcagCGCcttaggagatttgag	Fbw7 WD domain mutation
Rep.S	gccgccaaggatctgatg	qPCR for MCV origin sequence
Rep.AS	GAGAACCTGCGTGCAATC	qPCR for MCV origin sequence
pGEX-FWD.F	GGAAGAATTCGAACTCAAATCTCCTAAGG	FBW7 WD40 domain
pGEX-FWD.R	GGGAAGCTTTCACTTCATGTCCACATCAAAGTC	FBW7 WD40 domain
Fbw7.d278-324.F	GTGATAGAACCCCAGTTTCAATGCAAAGAAGAGGGGATTGATG	HA-FBW7ΔDF
Fbw7.d278-324.R	GGTTCATCAATCCCCTCTTCTTTGCATTGAAACTGGGGTTCTATCACTTG	HA-FBW7ΔDF
pGEXMCS.S	gatctgaaaacctgtatttccagagtggatccgaattcgtcgaccccgggtctagac	pGEX MCS
pGEXMCS.AS	TCGAGTCTAGACCCGGGGTCGACGAATTCGGATCCACTCTGGAAATACAGGTTTTCA	pGEX MCS

Table S3. Plasmids used in this study.

Table S2. Plasmids used in this study.			
**Plasmid name**	**Kwun Lab plasmid #**	**Addgene #**	**Notes**
pcDNA.sT.wt	83	40201	sT wild-type expression
pcDNA.sT.5A	84		sT mutant expression
pcDNA.sT.5E	91		sT mutant expression
pcDNA.sT.5H	115		sT mutant expression
pcDNA.sT.5K	114		sT mutant expression
pcDNA.sT.5R	92		sT mutant expression
pcDNA.LT	70	40200	LT expression
Rc/CMV cyclin E	337	8963	Cyclin E overexpression
pCR2.1-MCV ori339(97)	113		MCV origin plasmid
pLVX EF-MCS Puro	27		Lentiviral expression, empty vector, Figure 6
pLVX EF ST WT Puro	5		Lentiviral sT expression, Figure 6
pLVX EF ST.L142A Puro	338		Lentiviral sT expression, Figure 6
pLVX EF ST.5A Puro	339		Lentiviral sT expression, Figure 6
pLVX EF ST.5E Puro	120		Lentiviral sT expression, Figure 6
pLVX EF ST.5H Puro	117		Lentiviral sT expression, Figure 6
pLVX EF ST.5K Puro	119		Lentiviral sT expression, Figure 6
pLVX EF ST.5R Puro	121		Lentiviral sT expression, Figure 6
pCGN HA-Fbw7 WT	68		Wild-type FBW7
pCGN HA-Fbw7ΔDF(d231-324) WT	97		Dimerization/F-box domain deleted (ΔDF), wild-type WD40, used for binding assays
pCGN HA-Fbw7ΔDF(d231-324) R465C	288		WD40 residue mutation identified in CLL, Figure 2
pCGN HA-Fbw7ΔDF(d231-324) G423V	289		WD40 residue mutation identified in CLL, Figure 2
pCGN HA-Fbw7ΔDF(d231-324) W425C	291		WD40 residue mutation identified in CLL and ATL, Figure 2
pCGN HA-Fbw7ΔDF(d231-324) A503V	292		WD40 residue mutation identified in CLL, Figure 2
pCGN HA-Fbw7ΔDF(d231-324) R479Q	298		WD40 residue mutation identified in CLL, Figure 2
pCGN HA-Fbw7ΔDF(d231-324) R505C	299		WD40 residue mutation identified in CLL and ATL, Figure 2
pCGN HA-Fbw7ΔDF(d231-324) W406R	314		WD40 mutant, Figure 2
pCGN HA-Fbw7ΔDF(d231-324) S462P	316		WD40 mutant, Figure 2
pCGN HA-Fbw7ΔDF(d231-324) H468R	317		WD40 mutant, Figure 2
pCGN HA-Fbw7ΔDF(d231-324) D527G	318		WD40 mutant, Figure 2
pCGN HA-Fbw7ΔDF(d231-324) W486*	324		WD40 mutant, Figure 2
pCGN HA-Fbw7ΔDF(d231-324) N392S	322		WD40 mutant, Figure 3
pCGN HA-Fbw7ΔDF(d231-324) T416A/V418F	320		WD40 mutant, Figure 3
pCGN HA-Fbw7ΔDF(d231-324) C493G	306		WD40 mutant, Figure 3
pCGN HA-Fbw7ΔDF(d231-324) L494F	307		WD40 mutant, Figure 3
pCGN HA-Fbw7ΔDF(d231-324) V375A/K377N	319		WD40 mutant, Figure 3
pCI Flag c-Myc	293		c-myc overexpression
pCMVtag2B-eGFP	40		Transfection control
pGEX-GST	277		Bacterial expression, empty GST vector, Figure 5
pGEX-GST-FWD	335		Bacterial FBW7 WD40 domain expression, Figure 5

Table S4. Antibodies used in this study.

Name		Host species/Clonality	Detection
MCPyV CM2B4	Santa Cruz Biotechnology Inc	Mouse mAb	MCV LT
GFP (D5.1)	Cell Signalling Technology	Rabbit mAb	GFP
c-Myc (9E10)	DSHB	Mouse mAb	c-Myc
Cyclin E (HE12)	Santa Cruz Biotechnology Inc	Mouse mAb	Cyclin E
Anti-MCPyV T-antigen Antibody (2T2)	Millipore, CM LAB	Mouse mAb	MCV LT, sT
Anti-MCPyV T-antigen Antibody (8E6)	CM LAB	Mouse mAb	MCV LT, sT
HA-Tag (C29F4)	Cell Signalling Technology	Rabbit mAb	FBW7 constructs
anti-alpha-Tubulin (12G10)	DSHB	Mouse mAb	alpha-tubulin
ANTI-FLAG (M2)	Sigma	Mouse mAb	
IRDye 800CW goat anti-mouse IgG	LI-COR		
IRDye 800CW goat anti-rabbit IgG	LI-COR		
IRDye 680LT goat anti-rabbit IgG	LI-COR		
IRDye 680LT goat anti-mouse IgG	LI-COR		

Supplementary Materials and Methods

Plasmids and cell lines

Codon-optimized, commercially synthesized MCV LT and sT antigen clones were previously described (Addgene 40201 for sT, Addgene 40200 for LT) [5]. All sT and FBW7 WD40 mutants (from pCGN HA-FBW7) [6] were generated by overlapping PCR mutagenesis. Primer sequences for the constructions are described in Supplementary Table 2. The MCV replication origin plasmid (Ori339(97)) [7], pCGN HA-FBW7, cyclin E (Addgene 8963) [8] and Flag-cMyc have been previously described [6]. For GST-FWD, pGEX-KG multiple cloning site was modified with primers (Supplementary Table 2) and EcoRI/HindIIII sites were used. Plasmids used for this study were listed in Supplementary Table 3. U2OS and 293 cells were cultured in DMEM with 10% premium grade fetal bovine serum (FBS) (Seradigm). NIH3T3 cells were maintained in Dulbecco's Modified Eagle Medium (DMEM) with 10% Bovine calf serum (Seradigm). Cell lines were authenticated by short tandem repeat (STR) DNA profiling and tested for mycoplasma contamination prior to experimentation.

Structural analysis and docking modeling

PDB structure 2OVQ, 1P22, 4N14 were used [9-11] for WD40 domain analysis (Fig. 1). The model of MCV sT structure was generated using the I-TASSER server [12] based on SV40 sT homolog structures (PDB ID: 2PF4, 2PKG) [6]. Ribbon and sphere representations as well as structural alterations were achieved with the programs PyMOL and UCSF Chimera10. LSD mutations were generated via the rotamers function showing different rotameric states. Selected positioning was based on their highest probability and proximity to other residues. Electrostatic coloring of structural surfaces was based on the electrostatic potential via the Coulombic preset. The ClusPro server (https://cluspro.org) is used a tool for sT-FBW7 docking modeling [6].

Immunoblotting and Antibodies

Cells were lysed in IP buffer (50 mM Tris-HCl (pH 7.4), 150 mM NaCl, 1% TritonX-100, 1 mM PMSF, 1 mM benzamidine) and sonicated whole cell lysates were used for direct immunoblotting. Primary antibodies were incubated overnight at 4°C, followed by 1 h secondary antibody incubation at RT. All signals were detected using quantitative Infrared (IR) secondary antibodies (IRDye 800CW goat anti-mouse, 800CW goat anti-rabbit, 680LT

goat anti-rabbit IgG, 680LT goat anti- mouse IgG) (LI-COR). Signal intensities were analyzed using a laser-scanning imaging system, Odyssey CLX (LI-COR). Antibodies used for this study were listed in Table S4.

MCV Origin Replication by quantitative PCR Analysis

The MCV replication origin assay has been previous described [10]. 293 cells were transfected with expression vector (LT/sT) and pMCV-Ori339(97) using Lipofectamine 3000 (Invitrogen) in 12-well plates. Because mutations in the LSD cause to reduced expression levels of sT mutants, the optimal amount of sT mutant DNA to use in the transfection was adjusted to obtain comparable protein expression in each experiment. Episomal DNA was collected by salt-precipitation at 48 h post transfection. One µg of DNA was digested with DpnI, then 100 ng of digested DNA was subjected to qPCR. qPCR was carried out with PowerUp^{TM} SYBR Green Master Mix (Applied Biosystems) using a StepOnePlus Real-Time PCR system (Applied Biosystems) according to the manufacturer's protocol. Primer sequences used for both MCV origin detection are described in Table S2.

Co-immunoprecipitation (co-IP) assays

Cells were lysed in IP buffer (50 mM Tris-HCl (pH 7.4), 150 mM NaCl, 1% Triton X-100) freshly supplemented with 1 mM phenylmethylsulfonyl fluoride (PMSF), and 1 mM benzamidine. Lysates were incubated at 4°C overnight with 20 µl 50% slurry of anti-HA Agarose beads (Pierce) completely equilibrated with IP buffer. Beads were washed with IP buffer and high salt IP washing buffer (50 mM Tris-HCl (pH 7.4), 500 mM LiCl). Beads were resuspended in 2xSDS loading buffer, and all proteins were separated by SDS-PAGE (4-20% CriterionTM TGXTM precast gradient protein gels) followed by immunoblotting to detect interacting proteins. For all co-immunoprecipitation experiments, we used a FBW7ΔDF construct for wild-type (WT) WD40 expression (deletion of both dimerization and F-box domains that prevent SCF recruitment and thus uncouple substrate binding from its turnover) [13].

GST pulldown assay

For prey proteins, transfections were performed with Lipofectamine 3000 reagents (Life Technologies) using wild-type MCV sT and mutants sT (5A, 5E, 5H, 5K, 5R) constructs. Two days after transfection, 293 cells were lysed with immunoprecipitation lysis buffer and pre-cleared. GST-FWD plasmid was transformed into E. coli

Rosetta(DE3)pLysS strain (Novagen) for FBW7 WD40 domain expression. The WD40 protein was purified using glutathione-Sepharose 4B (GE Healthcare, Cat#17-0756-01) according to the manufacturer's protocol. Purified bait protein (GST-FWD) was coupled to beads and beads were blocked with BSA to prevent non-specific binding. Pulldown was conducted using glutathione-Sepharose 4B at 4°C overnight (GE Healthcare, Cat#17-0756-01). Beads from pulldowns were washed extensively with IP and LiCl wash buffers and eluted with an equal volume of 2XSDS loading buffer by heating at 95°C for 10 min. Western blots were carried out as described above, with mouse 8E6 Ab (1:1000) and IRDye 800CW goat anti-mouse (1:5000; LI-COR Biosciences).

Proximity ligation assay (PLA)

PLA was performed using Duolink assay kit (Sigma-Aldrich) according to the manufacturer's instructions. U2OS or 293 cells were fixed onto coverslips with paraformaldehyde (4% in PBS) for 5 min at room temperature at 48 h post transfection. Cells were permeabilized with PBS/0.1% Triton X-100 for 10 min. After three PBS washes, the cells were incubated with blocking solution for 1 h at 37°C followed by primary corresponding antibody pairs for 2 h at 37°C. The cover slips were washed twice for 5 min with buffer A, followed by incubation of the PLA probes (secondary antibodies against two different species (rabbit and mouse) bound to two oligonucleotides: anti-mouse MINUS and anti-rabbit PLUS). After two washes with buffer A, the probes were ligated by incubating a diluted ligase for 30 min at 37°C. Then, amplification step was achieved by incubating polymerase enzyme along with nucleotides for 100 min at 37°C. Cells were counterstained with DAPI (ThermoScientific) using a 0.1 μg/ml solution for 3 min at room temperature and mounted onto slides. Primary antibodies were utilized at optimized concentrations with HA-Tag (C29F4) rabbit mAb (1:2500), c-Myc (9E10) mouse mAb (1:2000), cyclin E (HE12) mouse mAb (1:2000), and 2T2 (1:2000) (Millipore). Fluorescence micrographs were collected by a REVOLVE4 fluorescent microscope (Echo Laboratories). The quantification of interactions detected by the PLA was performed using the public domain image processing program ImageJ. U2OS (>34) and 293 (>250) cells from each population were analyzed to quantify counted puncta. DAPI and TRITC channels were separated in order to quantify cell number and puncta count independently. For the DAPI channel images, a threshold was set to identify the nucleus and these images were converted to a binary image (black = 0 and white = 255). To ensure separation of the overlapping nuclei, a "Watershed" function was utilized. The analyzed particle command was used to count the number of separate nuclei, using the measurement of the area of the smallest nuclei as a filter.

The region of interest was also set to exclude cells that were on the edge of the frame. Similar steps were used to enumerate the puncta count, with the exception of the PLA filter being set at “0.0003-infinity”. ImageJ was also used to determine the mean fluorescence intensity by quantification of the integrated density. All calculated integrated densities were normalized to the non-fluorescent cells. Differences between means (*p* value) were analyzed using a t-test with GraphPad Prism software.

Proximity ligation assay (PLA) Flow cytometry

PLA was performed using Duolink flowPLA detection kit (Sigma-Aldrich) according to the manufacturer's instructions. Primary antibodies were utilized at optimized concentrations with HA-Tag (C29F4) rabbit mAb (1:500), c-Myc (9E10) mouse mAb (1:500), and 2T2 (1:500) (Millipore). Cells were washed twice in PBS with centrifugation (350x g, 5 min) and then analyzed by flow cytometry on a 16-color BD LSR Fortessa. The acquired data were analyzed using FlowJo software (Tree Star, Ashland, OR, USA).

References

1 Close V, Close W, Kugler SJ, Reichenzeller M, Yosifov DY, Bloehdorn J *et al*. FBXW7 mutations reduce binding of NOTCH1, leading to cleaved NOTCH1 accumulation and target gene activation in CLL. *Blood* (Article) 2019; 133: 830-839.

2 Aydin IT, Melamed RD, Adams SJ, Castillo-Martin M, Demir A, Bryk D *et al*. FBXW7 mutations in melanoma and a new therapeutic paradigm. *J Natl Cancer Inst* 2014; 106: dju107.

3 Yeh CH, Bellon M, Pancewicz-Wojtkiewicz J, Nicot C. Oncogenic mutations in the FBXW7 gene of adult T-cell leukemia patients. *Proc Natl Acad Sci U S A* 2016; 113: 6731-6736.

4 Yeh CH, Bellon M, Nicot C. FBXW7: a critical tumor suppressor of human cancers. *Mol Cancer* 2018; 17: 115.

5 Shuda M, Kwun HJ, Feng HC, Chang Y, Moore PS. Human Merkel cell polyomavirus small T antigen is an oncoprotein targeting the 4E-BP1 translation regulator. *Journal of Clinical Investigation* (Article) 2011; 121: 3623-3634.

6 Kwun HJ, Shuda M, Feng H, Camacho CJ, Moore PS, Chang Y. Merkel Cell Polyomavirus Small T Antigen Controls Viral Replication and Oncoprotein Expression by Targeting the Cellular Ubiquitin Ligase SCFFbw7. *Cell Host & Microbe* (Article) 2013; 14: 125-135.

7 Kwun HJ, Guastafierro A, Shuda M, Meinke G, Bohm A, Moore PS *et al*. The minimum replication origin of merkel cell polyomavirus has a unique large T-antigen loading architecture and requires small T-antigen expression for optimal replication. *J Virol* 2009; 83: 12118-12128.

8 Hinds PW, Mittnacht S, Dulic V, Arnold A, Reed SI, Weinberg RA. Regulation of retinoblastoma protein functions by ectopic expression of human cyclins. *Cell* 1992; 70: 993-1006.

9 Hao B, Oehlmann S, Sowa ME, Harper JW, Pavletich NP. Structure of a Fbw7-Skp1-cyclin E complex: multisite-phosphorylated substrate recognition by SCF ubiquitin ligases. *Mol Cell* 2007; 26: 131-143.

10 Wu G, Xu G, Schulman BA, Jeffrey PD, Harper JW, Pavletich NP. Structure of a beta-TrCP1-Skp1-beta-catenin complex: destruction motif binding and lysine specificity of the SCF(beta-TrCP1) ubiquitin ligase. *Mol Cell* 2003; 11: 1445-1456.

11 Sackton KL, Dimova N, Zeng X, Tian W, Zhang M, Sackton TB *et al*. Synergistic blockade of mitotic exit by two chemical inhibitors of the APC/C. *Nature* 2014; 514: 646-649.

12 Roy A, Kucukural A, Zhang Y. I-TASSER: a unified platform for automated protein structure and function prediction. *Nat Protoc* 2010; 5: 725-738.

13 Welcker M, Larimore EA, Swanger J, Bengoechea-Alonso MT, Grim JE, Ericsson J *et al*. Fbw7 dimerization determines the specificity and robustness of substrate degradation. *Genes Dev* 2013; 27: 2531-2536.
